# Is the ovarian reserve influenced by vitamin D deficiency and the dress code in an infertile Iranian population?

**DOI:** 10.1186/s13048-018-0435-7

**Published:** 2018-07-24

**Authors:** Soheila Arefi, Gholamreza Khalili, Homa Iranmanesh, Fattaneh Farifteh, Ahmad Hosseini, Human M. Fatemi, Barbara Lawrenz

**Affiliations:** 1 Avicenna Research Institute, ACECR, Tehran, Iran; 2 Bahman Hospital Infertility Center, North Iran Zamin St, Shahrak Gharb, Tehran, Iran; 3 Givar Infertility Center, Tehran, Iran; 4 Department of Epidemiology and Reproductive Health, Reproductive Research Center, Royan Institute for Reproductive Biomedicine, ACECR, Tehran, Iran; 5 Sarem Infertility Center, Sarem Women’s Hospital, Shahrak Ekbatan, Tehran, Iran; 6 Cellular and Molecular Biology Research Center, Shahid Beheshti University of Medical science, Arabi Ave, Daneshjoo Blvd, Velenjak, Tehran, Iran; 7 IVF department, IVI RMA Middle-East Fertility Clinic, Abu Dhabi, UAE; 8 Women’s university hospital Tuebingen, Tuebingen, Germany

**Keywords:** Dress code, Vitamin D deficiency, Ovarian reserve, Skin-type

## Abstract

**Background:**

In the recent years, vitamin D has become a topical subject and a focus of research not only in reproductive medicine but across many medical disciplines. In reproductive medicine, studies have identified an association between vitamin D status in women and ovarian reserve. In humans, exposure of the skin to sunlight is the main important source of vitamin D. A dress code of wearing concealing clothing is a risk factor for vitamin D deficiency. The objective of this prospective observational study was to evaluate the correlation between vitamin D deficiency and ovarian reserve in a population of infertile women in Iran. As part of the basic fertility assessment of study participants, blood tests were taken to measure vitamin D concentration and transvaginal ultrasound scans were performed on day 2–5 of the cycle to determine antral follicle count (AFC). All study participants were assessed by a reproductive medicine specialist and consultant dermatologist to classify their skin types according to the Fitzpatrick classification. In addition, the dress code of each study participant was recorded noting the percentage of exposed skin not covered by concealing clothing.

**Results:**

189 infertility patients were included in this study. The mean concentration of vitamin D in this study population was 15.46 ng/ml, indicating severe vitamin D deficiency. A statistically significant negative correlation between age and vitamin D (*p* = 0.008) and age and AFC (*p* = 0.001) was identified. This study revealed a highly significant correlation between vitamin D concentrations and AFC (*p* < 0.001).

**Conclusions:**

A concealing dress code is an independent risk factor for vitamin D deficiency due to a lack of skin exposure to sunlight. Our study suggests that the so caused severe vitamin D deficiency may play a crucial role in reduced ovarian reserve in the herein described group of an infertile female Iranian population.

## Background

In recent years, vitamin D has become a topical subject and a focus of research in several medical disciplines.

Vitamin D is widely recognized to facilitate absorption of calcium and promote bone growth. Vitamin D deficiency in children may result in a condition known as rickets incurring an increased risk of fractures and deformity. In adults, vitamin D deficiency may result in osteomalacia carrying the same risks of fracture and deformity. In addition to its role in bone health, it appears that vitamin D deficiency is also involved in the pathogenesis of many chronic diseases such as autoimmune diseases, inflammatory bowel disease, infections, immune deficiency, cardiovascular diseases, cancer and neurocognitive disorders [1].

As vitamin D receptors are widely found in reproductive tissues, including the ovaries and the endometrium, an important role of vitamin D in human reproduction has been suggested. Vitamin D status has been suggested to be associated with ovarian reserve and with the outcome of in vitro fertilization (IVF) treatments as well as with features of polycystic ovarian syndrome (PCOS) and endometriosis. In men, associations with semen quality and sperm count, motility and morphology had been described [2]*.*

Vitamin D belongs to a group of fat-soluble vitamins and is responsible for the intestinal absorption of calcium, magnesium, phosphate, zinc and iron. The most important forms of Vitamin D are vitamin D3 (cholecalciferol) and vitamin D2 (ergocalciferol) [3]. According to recent studies, vitamin D deficiency is defined as serum-levels of 25(OH)D below 20 ng/ml. The importance of vitamin D deficiency is underlined by the fact, that an estimated 1 billion people worldwide are vitamin D deficient and deficiencies can be found in all ethnicities and age groups [4]. Therefore vitamin D deficiency is the most common vitamin deficiency worldwide in both, children and adults [5].

Dietary sources of vitamin D alone are not sufficient to maintain normal vitamin D levels.

The most important source of vitamin D for humans is exposure to sunlight, particularly ultraviolet B rays. Several factors influence vitamin D production following sun exposure. These factors include age, skin color, degree of exposed skin, length of time exposed to sunlight, geographic location, time of year, time of day, cloud cover, smog, dust or haze.

The countries of the Middle East have the highest vitamin D deficiency rates in the world, ranging from 67% in Iran, 55–83% in Jordan, 84% in Lebanon up to 90% in Saudi Arabia [6].

Recently, several studies evaluated the influence of vitamin D on the ovarian reserve [7–9]. In contrast to the herein presented study, the participants consisted mainly of women of Caucasian ethnicity and were not subjected to imposed female dress codes. Our study is unique in that our participants are of Iranian ethnicity and wear concealing clothing, a dress code imposed by both, religious and social values.

The aim of this study was to evaluate the vitamin D status in an infertile population in Iran and to correlate vitamin D concentrations with parameters such as female age, skin type, degree of skin concealment and antral follicle count (AFC) as parameter of the ovarian reserve.

## Methods

This observational prospective study was performed in 2 independent private infertility centers in Iran. Patients, referred to the fertility centers with both, primary and secondary infertility, were considered eligible for inclusion in the study. Exclusion criteria included any factors which may adversely affect ovarian reserve such as a history of endometriosis, history of previous pelvic surgery, smokers and recreational drug users, history of chemotherapy or pelvic radiation and a history of hormonal treatment in the previous 6 months. Polycystic ovarian syndrome as defined by the Rotterdam-criteria [10] was also included as an indication for exclusion from the study. The intake of calcium, vitamin D or multivitamin supplements in the previous 3 months or a history of poor ovarian response according to the Bologna criteria [11] in a previous ART-treatment were also exclusion factors.

All study participants signed an informed consent agreement. Blood for vitamin D concentrations were measured via venipuncture and was taken together with other blood tests, deemed necessary for evaluation of the existing infertility and further treatment planning. Due to the additional costs which would have been created by performing AMH-testing and which would have to be covered by the patients, no routine AMH-test was performed. Transvaginal 2D ultrasound of the pelvis was performed on day 2–5 of the cycle to exclude pelvic pathology and to determine AFC.

### Ultrasound for antral follicle count measurement

All patients were scanned by transvaginal 2D ultrasound on day 2–5 of the cycle. The ultrasound-scans were performed by one designated reproductive medicine specialist in each center in order to reduce the inter-observer variability. The two involved investigators established a common ultrasound technique for the AFC measurement prior of commencing the study in order to avoid any bias through different techniques.

All study participants had a transvaginal scan performed by the same investigator for intra-observer or inter-observer reliability, using a vaginal probe (Honda 5–9 MHZ, HS-2600, Japan). The optimal probe program, providing the best ultrasound image, was used in both fertility centers and the settings were maintained throughout the duration of the ultrasound evaluation. Each patient was asked to empty the bladder and was scanned in a Lloyd Davies position to ensure free manipulation of the transvaginal transducer. Ultrasound assessment of the pelvis excluded pelvic pathology. The ovaries were visualized in the longitudinal plane and the number of antral follicles measuring 2–10 mm in diameter within each ovary was counted as the transducer was moved from one side of the ovary to the other. Two scans were obtained for each ovary and in case of any discrepancy between these two values, a third measurement was done to confirm the final value.

### Skin-type

The skin-types of all study participants was evaluated and classified according to the different skin type groups described by the Fitzpatrick classification [12] by a reproductive specialist and a consultant dermatologist.

Type I-II Fitzpatrick classification – abbreviated in this study as F1:

- Type I (scores 0–6) always burns, never tans (pale white; blond or red hair; blue eyes; freckles)
- Type II (scores 7–13) usually burns, tans minimally (white; fair; blond or red hair; blue, green, or hazel eyes)

Type III – IV 3–4 Fitzpatrick classification – abbreviated in this study as F2:

- Type III (scores 14–20) sometimes mild burn, tans uniformly (cream white; fair with any hair or eye color)
- Type IV (scores 21–27) burns minimally, always tans well (moderate brown)

Type V - Fitzpatrick classification – abbreviated in this study as F3:

- Type V (scores 28–34) very rarely burns, tans very easily (dark brown)

### Dress-code

According to the Iranian regulations, in any public place women must cover their heads with a headscarf, wear trousers (or a floor length skirt), and a long-sleeved tunic or coat that reaches to mid-thigh or knee. Depending on the amount of skin not covered by concealing cloths, patients were stratified into the dress code (DR) 1, 2 and 3. Patients with DR 1 had an amount of uncovered skin of 10 to 15%, patients in DR 2 between 5 and 10% and patients in group DR 3 below 5% of skin exposed to sunlight.

### Measurement of vitamin D-levels

Blood samples were taken to measure vitamin D concentrations within the first week following the initial consultation with a reproductive medicine specialist and at the same time of ultrasonographic assessment of the antral follicle count (AFC). Once the blood sample was taken for vitamin D concentration, the sample was centrifuged and frozen at − 20° Celsius. All blood samples for vitamin D concentrations were measured by the ELISA assay at one time to avoid inter-assay variability, using electrochemiluminescence binding assay (Roche’s test) and Cobas e immunoassay analyzers.

Vitamin D deficiency was defined as serum level of < 20 ng /ml 25hydroxy vitamin D [3].

### Statistical analysis

Data were analyzed by SPSS v.18 software (IBM, Chicago, IL, USA). The quantitative measurements were described by using the mean and the standard deviation (SD). For nominal variables the relative frequency, using percentages, were reported. For comparison of means among groups, analysis of variance and LSD post hoc test was used.

## Results

A total of 189 patients have been included in this prospective observational study. 87.7% (*n* = 166) of the patients attended the clinics due to primary infertility. The mean BMI of the patients was 26.70 kg/m^2^ with a range of 17.9–35.5 kg/m^2^.

According to the Fitzpatrick classifications, 50 patients (26.4%) were categorized as Fitzpatrick I-II (F1), 89 (47.1%) as Fitzpatrick III-IV (F2) and 50 (26.4%) as Fitzpatrick V (F3).

The patients were also classified according to dress code DR1, DR2 and DR 3 as previously described with the following distribution, 110 (58.2%), 30 (15.9%) and 49 (25.9%) respectively.

The mean age of the study group was 32.21 years and for the groups F1, F2 and F3 31.76 years, 32.40 years and 32.32 years respectively. The mean age of the patients in the different groups classified according to dress-code groups were as follows: DR1: 31.97 years, DR2: 33.07 years and DR3: 32.22 years.

The mean vitamin D level for the total study group was 15.46 ng/ml. In group F1 mean vitamin D level was 16.39 ng/ml, in group F2 14.50 ng/ml and in group F3 16.23 ng/ml. The mean Vitamin D level for groups DR1, DR2 and DR3 were 16.15 ng/ml, 13.66 ng/ml and 14.21 ng/ml, respectively.

The mean AFC count in all patients was 10.84. Patients in group F1 had a mean AFC of 10.14, group F2 a mean AFC of 11.20 and group F3 a mean AFC of 10.90. In the dress-code groups the results were: DR1: 10.59, DR2: 9.87 and DR3: 12.00, respectively. Table 1 provides the summary of the results.

**Table 1:** Summary of the results for the means, ranges and standard deviations for the following parameters: age, vitamin D concentrations and AFC in the different skin-type- and dress-code-groups

Groups		Total group	F1	F2	F3	DR1	DR2	DR3	Significance
Number of patients and %		189 (100%)	50 (26.4%)	89 (47.1%)	50 (26.4%)	110 (58.2%)	30 (15.9%)	49 (25.9%)	n.a.
Age (years)	Mean	32.21	31.76	32.4	32.32	31.97	33.07	32.22	n.s.
	Range	21–42	21–38	23–43	21–38	21–39	27–42	26–40	
	SD	3.64	3.63	3.69	3.58	3.67	3.83	3.44	
Vitamin D level (ng/ml)	Mean Vit. D level (ng/ml)	15.46	16.39	14.50	16.23	16.15	13.66	14.21	n.s.
	Range	2.5–73	2.5–43.8	2.5–73	4–48	2.5–73	2.5–48	4.0–44	
	SD	11.5	11.01	11.32	11.05	11.91	10.54	11.08	
AFC	Mean AFC	10.84	10.14	11.20	10.90	10.59	9.87	12.00	n.s.
	Range	1–31	2–29	2–31	1–30	1–31	2–29	2–29	
	SD	6.63	6.45	7.06	6.06	6.43	7.54	6.37	

*n.a.* not applicable, *n.s.* not significan, *SD* Standard deviation

The correlation of the data of all study patients between the parameters age, vitamin D concentrations and AFC showed a significant negative correlation between age and vitamin D (*p* = 0.008), age and AFC (*p* = 0.001) and importantly, a highly statistically significant correlation between vitamin D and AFC (*p* < 0.001).

No significant differences were found between and within the different skin-type groups for age (*p* = 0.59), vitamin D (*p* = 0.56) and AFC (*p* = 0.66). The groups of different dress-codes were also found to have no significant differences between and within the group for the parameters age (*p* = 0.34), Vitamin D (*p* = 0.32) and AFC (*p* = 0.31). Also no correlations were found between vitamin D and BMI (*p* = 0.968) and vitamin D and the type of infertility (*p* = 0.451). The performance of multiple comparisons in the groups age, vitamin D and AFC within the different skin types as well as within the different dress codes did not show any significant differences.

## Discussion

The presence of vitamin D receptors in reproductive tissues [13, 14] and the fact that in animal studies impaired folliculogenesis [15] was found in rats with compromised expression of vitamin D receptors suggests, that vitamin D deficiency may play an important role in determining the ovarian reserve. It was previously described that 1.25-dihydroxyvitamin D3 alters Anti-Mullerian-Hormone (AMH) sensitivity in human granulosa cells by inhibiting the expression of the AMH receptor (AMHR-II). Due to a reduced AMH sensitivity, more follicle might reach terminal maturation and ovulation, thereby influencing the ovarian reserve [16]. This possible interaction between the ovarian reserve and vitamin D is underlined by a correlation of seasonal fluctuations in serum AMH with seasonal changes in the vitamin D levels [17]. Due to this correlation, Irani et al. [7] even considered vitamin D to be a clinically useful marker of the ovarian reserve and suggested to include vitamin D into the routine workup in patients with infertility.

The herein presented study findings result from a prospective observational study in 189 female infertility patients in Iran, who are obliged to wear a specific dress code. The results demonstrate clearly a highly significant correlation (*p* < 0.001) between vitamin D deficiency and a reduced ovarian reserve. In this setting of self-payer patients, ovarian reserve was assessed by the antral follicle count (AFC), which is an established parameter to evaluate the ovarian reserve [18].

The most commonly used parameters for ovarian reserve testing are FSH (Follicle-stimulating-hormone), AMH (Anti-Muellerian-Hormone) and the antral follicle count. Nowadays, FSH is regarded as a parameter with poor sensitivity towards the detection of a diminished ovarian reserve and FSH testing has several major limitations including significant inter-cycle and intra-cycle variability and elevated FSH levels are almost only found in patients with an existing diminished ovarian reserve [19]. AMH is produced by the granulosa cells of small and large preantral and small antral follicles, therefore reflecting the primordial follicle pool. Whereas it was suggested that AMH is relatively stable throughout the cycle [20, 21], recent publications demonstrated significant fluctuations within a menstrual cycle [22, 23]. The antral follicle count (AFC) is calculated as the sum of follicles in both ovaries in the early follicular phase (day 2–4) of the menstrual cycle. The assessment can be performed in the context of the primary ultrasound evaluation of the internal genital organs and it provides an immediate result, has a good inter-cycle reliability and, if performed by experienced and a limited number of investigators, a good inter-observer reliability [24]. Several studies have shown that AFC and AMH are both good predictors of the ovarian response in the context of hormonal stimulation for ART (assisted reproductive techniques) treatment [25, 26], with each method having special advantages, disadvantages and limitations.

Studies to date, investigating the role of vitamin D on the ovarian reserve are conflicting and our results are in contrast to the findings from the recently published studies of Fabris et al. [8] and Drakopoulos et al. [9] who could not find a correlation between the ovarian reserve parameters AFC / AMH and vitamin D deficiency.

The contradictory finding between the studies may be attributed to the different ethnicities of the study population, having different socio-cultural religious habits and different dress-codes. Drakopoulos et al. [9] evaluated women with Caucasian ethnicity, who have been treated in an infertility center due to primary / secondary infertility in Belgium. The study of Fabris et al. [8] included only oocyte donors, who are usually young and healthy and don’t have any infertility problems. The ethnicity of the study population is not revealed. However, their data are derived from an oocyte-donation-program based at a Spanish IVF-clinic. Therefore it can be assumed that their study patients had Caucasian ethnicity as well. In contrast to those mentioned studies, the herein described study population presents firstly a completely different ethnicity and secondly around 40% of the study population is dressed in a dress-code, which leaves less than 90% of the skin uncovered, thereby limiting the skin-exposure to the sun dramatically.

The Middle East countries have the highest vitamin D deficiency rates in the world and different factors contribute to low sun exposure and low vitamin D levels in women of reproductive age in these countries [27]. One of the most important factors leading to a high rate of vitamin D deficiency is the dress code of wearing concealing clothing due to social and religious habits [28]. The concealing dress code has been found to be an independent prognostic factor of vitamin D deficiency in Lebanese women, confirming the significance of sunlight exposure in vitamin D synthesis [28] and differences observed in vitamin D deficiency rates between Christian and Muslim women in Middle East highlight the importance of the dress code [29].

Skin-type is an important factor in determining the time needed for sufficient vitamin D synthesis via sunlight exposure. Persons with a skin-type classification I or II achieve maximal vitamin D photosynthesis rapidly after approximately 2–8 min of midday spring or summer sun exposure in New York or Boston and only slightly longer in Alaska or Scandinavia. Persons with phototype III skin produce vitamin D at similar rates to persons with phototype I or II skin after a first UV exposure. Persons with skin-type IV have high epidermal melanin content and therefore photosynthesize relatively limited amounts of vitamin D because of UV absorption by melanin [30]. Other authors deem the exposure of arms and legs for 5 to 30 min between 10 a.m. and 3 p.m. twice a week for sufficient to achieve adequate vitamin D levels [31].

However, in most Middle-East Islamic populations, girls start to wear concealing clothing with the onset of puberty or even earlier, leading to long-lasting non-exposure of the skin to the sun and therefore to long-lasting Vitamin D deficiencies.

Evaluation of the influence of the skin-type and dress-code on the Vitamin D deficiency was performed in a subgroup-analysis and no significant correlations between the different skin-types / dress-codes and vitamin D deficiency were found. Due to wearing concealing clothing with coverage of most of the body surface, the skin-type is of negligible importance. Absence of a correlation between the different dress-codes and vitamin D deficiency is explained by the fact, that even the group with the least skin coverage (10–15% at most of body skin surface which could be exposed to the sun) is still far below the required 20%.

Our study population is characterized by a severe vitamin D deficiency with 71.4% (135 patients) of the patients being vitamin D deficient and a mean vitamin D level in the study group of 15.48 ng/ml. In contrast, the mean vitamin D levels are not mentioned in the studies of Drakopoulos and Fabris [8, 9]. In addition, less than 50% of their study populations were vitamin D deficient (30 and 18% respectively). Therefore the severity of vitamin D deficiency in their studies, when compared to our study, was less and may be the reason why no correlation between vitamin D deficiency and the ovarian reserve parameters was identified.

## Conclusions

The herein presented data may indicate, that severe and long-lasting vitamin D deficiency, caused by inadequate sun-exposure to the skin, could be a cause of the reduced ovarian reserve in the herein investigated group of infertile female Iranian patients. Future studies are required to investigate further the interaction between dress-code, vitamin D and fertility.
